# Semantic similarity and machine learning with ontologies

**DOI:** 10.1093/bib/bbaa199

**Published:** 2020-10-13

**Authors:** Maxat Kulmanov, Fatima Zohra Smaili, Xin Gao, Robert Hoehndorf

**Affiliations:** King Abdullah University of Science and Technology; King Abdullah University of Science and Technology; Computational Bioscience Research Center and lead of the Structural and Functional Bioinformatics Group at King Abdullah University of Science and Technology; King Abdullah University of Science and Technology

**Keywords:** machine learning, semantic similarity, ontology, knowledge representation, neuro-symbolic integration

## Abstract

Ontologies have long been employed in the life sciences to formally represent and reason over domain knowledge and they are employed in almost every major biological database. Recently, ontologies are increasingly being used to provide background knowledge in similarity-based analysis and machine learning models. The methods employed to combine ontologies and machine learning are still novel and actively being developed. We provide an overview over the methods that use ontologies to compute similarity and incorporate them in machine learning methods; in particular, we outline how semantic similarity measures and ontology embeddings can exploit the background knowledge in ontologies and how ontologies can provide constraints that improve machine learning models. The methods and experiments we describe are available as a set of executable notebooks, and we also provide a set of slides and additional resources at https://github.com/bio-ontology-research-group/machine-learning-with-ontologies.

## Introduction

Machine learning methods are now applied widely across life sciences to develop predictive models [1]. Domain-specific knowledge can be used to constrain search and find optimal or near-optimal solutions faster, or to find better solutions; this observation has led Feigenbaum in 1977 to suggest that the power of Artificial Intelligence systems lies in the domain-specific knowledge they encode and are able to exploit, leading to the paradigm that ‘in the knowledge lies the power’ [2].

In the life sciences, domain-specific knowledge is often encoded in ontologies and in the database and knowledge base that use ontologies for annotation. Hundreds of ontologies have been developed, spanning almost all domains of biological and biomedical research. The main features biomedical ontologies provide are controlled vocabularies for characterizing biological phenomena and as formalized knowledge bases that formally describe the phenomena within a domain and link them to other related domains [3]. For example, phenotype ontologies are used for characterizing the phenotypes observed in a variety of model organism databases [4–7] as well as in human genetics [8, 9], and these ontologies provide a controlled set of classes, their labels and definitions for the purpose of annotating the phenotypes observed in conditions recorded in databases. Moreover, phenotype ontologies are also interlinked with other ontologies through the use of formal axioms and can be used to relate the phenotype observations to biological functions, anatomical locations, developmental stages or chemical substances [10, 11]. The majority of biomedical ontologies are formalized using the Web Ontology Language (OWL) [12], a language based on Description Logic (a decidable fragment of first order predicate logic). OWL comes with an explicit semantics that defines how statements made in OWL constrain the world in which these statements are interpreted—the ‘models’ in which these statements are true.

The background knowledge contained in ontologies can be used in several ways. Some important applications include the automatic and consistent construction of ontologies based on axioms and querying domain knowledge or data associated with ontology classes using the axioms. Constructing ontologies based on axioms, and in particular referencing classes from other ontologies in these axioms [3, 13, 14], allows reuse of existing knowledge and enables verification of consistency (i.e. absence of contradictions). For example, an ontology of phenotypes can be constructed by referencing axioms in anatomy ontologies [4, 11] so that phenotype classes are structured consistently with anatomy ontologies. This also enables querying phenotypes based on anatomy ontologies; for example, phenotypes such as *cardiomyopathy* can be retrieved as phenotypes of the *heart* or of parts of the *cardiovascular system*.

The combination of classes from different ontologies in ontology axioms can also be used to induce background knowledge in predictive analysis; axioms can be used to expand or enrich features in machine learning or to constrain the search for a solution to an optimization problem. Expanding or enriching features may make information available to a machine learning model that it would not be able to access without relying on ontologies. For example, linking phenotypes such as *cardiomyopathy* to the anatomical structures that are affected (i.e. the *heart*) can create novel and direct associations with other datasets that do not otherwise exist. In the example of *cardiomyopathy*, the link to *heart* as the anatomical structure can be used to relate the phenotype to gene expression in *heart* tissue or in cardiomyocytes or to biological processes such as *heart development*. Such connections are given *a priori* through the axioms in phenotype, anatomy and cell-type ontology and do not need to be discovered from data.

The knowledge in ontologies may be used to constrain the search for solutions to an optimization problem and thereby finding a solution faster, finding a better solution or finding a solution that is generalized better. One example of such a constraint is the ‘true path rule’ that was originally proposed in the Gene Ontology [15], which states that if a gene product $G$ has the potential to be involved in a process $P_1$, and every process $P_1$ is a part of another process $P_2$, then $G$ must also be involved in $P_2$. This constraint is ‘hard’ in that it is not an empirical law or observation, but should hold in virtue of the definition of $P_1$ and $P_2$—it is *impossible* for $G$ to participate in $P_1$ but not $P_2$. For example, a gene product involved in *cardiac muscle tissue development* ( GO:0048738) must be involved in *heart development* ( GO:0007507) simply based on the definition of the two classes in the Gene Ontology.

With the rapid growth of methods to build predictive models in biology, in particular machine learning methods [16, 17], biomedical ontologies can now play a role in systematically providing domain knowledge to enable or improve the predictive models. It is a challenge to identify general ways in which ontologies, and their underlying formalisms based on OWL, can be combined with the modern machine learning and optimization methods that are becoming so widespread. This challenge is not only one of the researches in Artificial Intelligence but also a new challenge in Bioinformatics research as well due to the widespread use of ontologies and formalized knowledge bases in biology and biomedicine and the unique characteristics of biomedical ontologies such as their large size and the manually created axioms.

Here, we describe and review the state-of-the-art and recent advances in accessing and exploiting background knowledge in ontologies to build predictive models in biomedicine, including similarity-based predictions and machine learning models. We use as a starting point in our review more traditional semantic similarity measures applied to ontologies; semantic similarity measures are a method from Artificial Intelligence that can determine the similarity between two or more entities using the formalized background knowledge in ontologies. We continue to introduce unsupervised, representation learning methods on ontologies that generate ‘embeddings’ for entities in ontologies, and we show that these embeddings can be used like semantic similarity measures while additionally allowing to overcome some of their limitations. Third, we highlight methods that use ontologies as constraints in optimization problems or to design architectures of machine learning models. We continue by introducing a novel benchmark dataset for prediction of protein–protein interactions (PPIs) with ontologies and demonstrate the methods we discuss on this dataset. We also make all experiments available as executable notebooks which can be adopted to other use cases. We finish by reviewing some of the main limitations and future research directions for the combination of ontologies and machine learning.

## Fundamentals: axioms, graphs and knowledge graphs

An ontology is an ‘explicit specification of a conceptualization of a domain’ [18], i.e. an ontology is an artifact used to formally specify the intended meaning of a vocabulary within a domain. Ontologies contain domain knowledge, encoded in the form of axioms, natural language labels, synonyms, definitions and other types of annotation properties. The majority of ontologies in the life sciences are encoded using the OWL [12], a language that is a part of the Semantic Web stack [19] and based on Description Logics [20]. Description Logics enable a formal, machine-readable description of the types of entities within a domain and the relations in which they stand [20]. Syntactic constructs are assigned an interpretation in a mathematical structure that resembles a world in which these constructs are true. For example, if we want to assert that one class of entities $C$ is more specific than another class $D$, i.e. $C$ is a $D$, we can (syntactically) write $C \sqsubseteq D$ using the common syntax of Description Logics. Semantically, we interpret each class $C$ and $D$ as a set of entities $C^{\mathcal{I}}$ and $D^{\mathcal{I}}$ (coming from a domain $\Delta $) and say that $C \sqsubseteq D$ is true when $C^{\mathcal{I}} \subseteq D^{\mathcal{I}}$. Depending on the choice of $C^{\mathcal{I}}$ and $D^{\mathcal{I}}$, there are many (algebraic) structures in which $C \sqsubseteq D$ can be true. In general, $\cdot ^{\mathcal{I}}$ is an *interpretation* function that assigns symbols to their extension in an algebraic structure, and the structures in which a statement is true is called a model of the statement.

The semantics of logical languages gives rise to entailment; a statement $\phi $ is logically entailed by a set of statements $O$ if all the structures in which all statements in $O$ are true also make $\phi $ true. For example, the two statements $\{C \sqsubseteq D, D \sqsubseteq E\}$ entail the statement $C \sqsubseteq E$ (because no matter the choice of $C^{\mathcal{I}}$, $D^{\mathcal{I}}$ and $E^{\mathcal{I}}$, $C^{\mathcal{I}} \subseteq D^{\mathcal{I}}$ and $D^{\mathcal{I}} \subseteq E^{\mathcal{I}}$ implies $C^{\mathcal{I}} \subseteq E^{\mathcal{I}}$). The process of computing entailments—deduction or logical inference—plays a crucial role in using ontologies because it allows to automatically derive statements that are not explicitly asserted in a knowledge base and can also be used to detect whether a set of statements is contradictory. In general, from a finite number of axioms, an infinite number of additional statements (the ‘deductive closure’ of the axioms) is entailed, and from a set of contradictory axioms, all statements are entailed (and, consequently, a contradiction can be detected by testing for the entailment of two contradictory statements). While this property makes logic-based methods a powerful means for storing and reasoning about knowledge, it also makes it more challenging to fully exploit this knowledge in computational models due to the possibly infinite amount of statements that can be generated through entailments.

Although ontologies in OWL are primarily sets of axioms, many ontology-based analysis methods, including machine learning methods and semantic similarity measures, rely on generating some form of graph structures from the axioms in an ontology. There are several ways in which axioms can be used to generate a graph structure, and many can be formulated as computing entailments. An important ontology for generating graphs from biomedical ontologies is the OBO Relation Ontology [14] which provides a set of axiom patterns that must hold true for two classes if an edge between them should be created [21–23]. An axiom pattern is an axiom with variables for classes or individuals; $X \sqsubseteq Y$ is an axiom pattern in which $X$ and $Y$ are variables and if this statement is true for two classes $X$ and $Y$, an edge labels is-a should be created between them: $X \xrightarrow{\text{is-a}} Y$. More complex axiom patterns involve quantifiers, such as $X \sqsubseteq \exists \mbox{part-of}.Y$ which gives rise to the edge $X \xrightarrow{\text{part-of}} Y$. Axioms can also express disjointness between two classes such as $X \sqcap Y \sqsubseteq \bot $ based on which a disjoint edge can be created ($X \leftrightarrow{\textrm{disjoint}} Y$). For a conversion of axioms into nodes and edges to be generally applicable, it must be possible for it to be generated using entailments; for example, if $X \sqsubseteq \exists \mbox{part-of}.Y$ and $Y \sqsubseteq \exists \mbox{part-of}.Z$ are a part of an ontology, and the relation part-of is transitive ($\mbox{part-of} \circ \mbox{part-of} \subseteq \mbox{part-of}$), then $X \sqsubseteq \exists \mbox{part-of}.Z$ would be entailed and consequently a part-of edge between $X$ and $Z$ created ($X \xrightarrow{\text{part-of}} Y$).

The types and complexity of axiom patterns giving rise to edges is an active research area [24, 25], and the translation patterns that are used depend not only on the axioms but also on the algorithms that use the graphs generated from ontologies. For example, OWL2Vec [26] uses the set of transformation rules shown in Table 1 to transform syntactic axiom patterns into edges. Depending on the algorithm that uses the graph, these patterns can be applied to the asserted or entailed set of axioms.

**Table 1:** OWL2Vec rules for the projection of OWL axioms into an RDF graph. $Q$ is any quantifier ($\exists $, $\forall $, $\leq n$, $\geq n$, $= n$); $A$, $B$, $B_i$ and $C_i$ are named classes; $S_i$, $R_o$ and $R^{-}$ are object properties; $b$ is an individual name

**Condition 1**	**Condition 2**	**Edge**
$A \sqsubseteq Q R_o.D $ or $Q R_o.D \sqsubseteq A $	$ D \equiv B|B_1 \sqcap ... \sqcap B_n|B_1 \sqcup ... \sqcup B_n$	$A \xrightarrow{R_o} B$ or $A \xrightarrow{R_o} B_i\; \mbox{ for}\; i \in 1...n$
$Domain(R_o) = A$	$Range(R_o) = B$	$A \xrightarrow{R_o} B$
$A \sqsubseteq \exists R_o.\{b\}$	$b: B$	$A \xrightarrow{R_o} B$
$R_o = R^-$	$A \xrightarrow{R} B$ in the graph	$B \xrightarrow{R_o} A$
$S_1 \circ ... \circ S_n \subseteq R_o$	$ A \xrightarrow{S_1} C_1,..., C_n\xrightarrow{S_n} B$ in the graph	$A \xrightarrow{R_o} B$
$B \sqsubseteq A$		$B \xrightarrow{is-a} A$ , $A \xrightarrow{is-a^{-}} B$

The graphs generated from ontologies also interact with graph-based representations of data, in particular using the Resource Description Framework (RDF) [27]. Graphs in which nodes represent entities within a domain and edges represent the relations between the nodes are sometimes called *knowledge graphs* [28], and they correspond to a subset of the formalism underlying OWL in which only relations between individuals, and possibly certain axioms for relations, are considered. However, graph-based representations of the axioms in ontologies can also be considered knowledge graphs, in particular when both individuals and classes are included in the graph [24]. For example, a graph in which different types of interactions between proteins are expressed could be generated from relation assertions such as $\mbox{binds}(P_1, P_2)$ (indicating that protein $P_1$ binds $P_2$) where $P_1$ and $P_2$ are individuals and translate directly into an edge between nodes $P_1$ and $P_2$, or the graph can be generated from more complex axioms such as $P_1 \sqsubseteq \exists \mbox{coex}.P_2$ in which $P_1$ and $P_2$ are classes and all instances of $P_1$ are co-expressed with some instance of $P_2$. Similarly, a statement that a protein $P$ has a function $F$ can be a direct relation assertion $\mbox{hasFunction}(P,F)$ (which is, for example, used in the RDF representation of the UniProt knowledge base [29] and translates directly into a corresponding edge between $P$ and $F$), or a more complex axiom such as $P \sqsubseteq \exists \mbox{hasFunction}.F$ [30]. The latter representation gives rise to entailments directly when combined with an axiom such as $F \sqsubseteq F^{\prime}$, while the first representation needs additional rules to achieve the same result.

**Figure 1:**
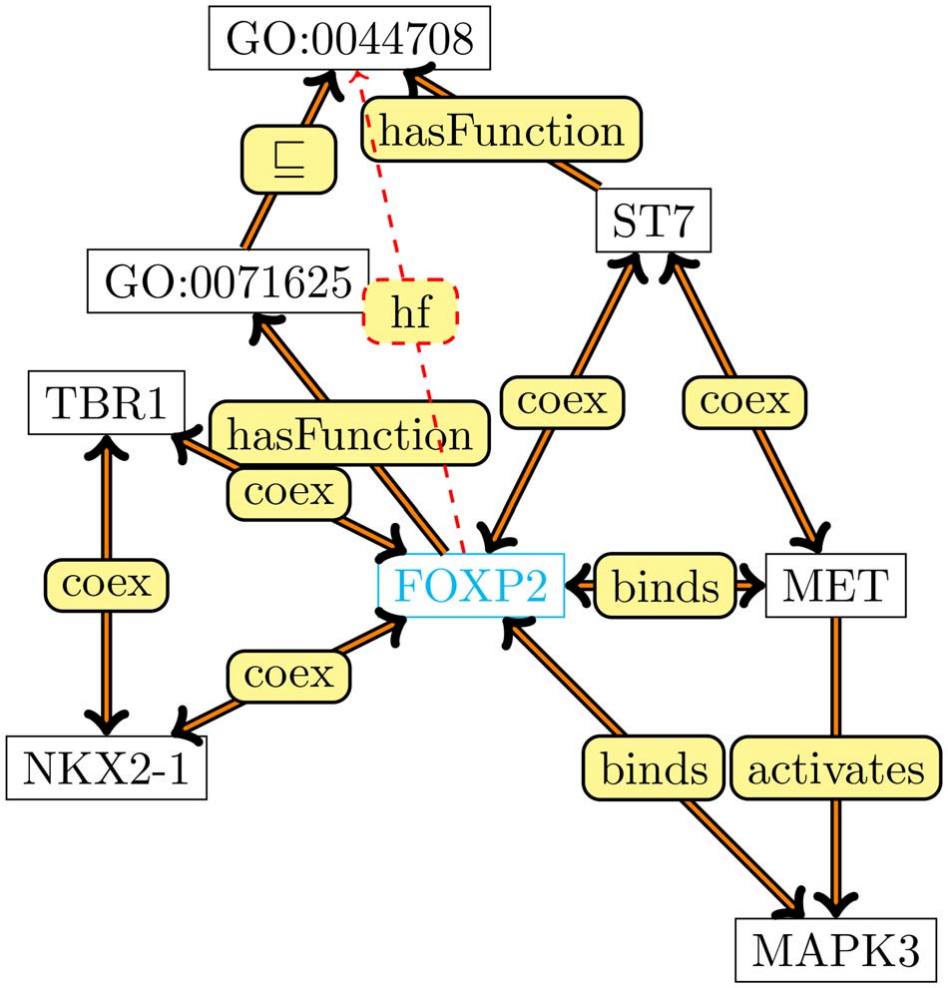
A knowledge graph centered around protein–protein interactions and functions of FOXP2.

Figure 1 shows a graph in which interactions between proteins, the associations between proteins and their functions and some axioms from the Gene Ontology are included. There are several ways in which such a graph could have been represented in OWL and then converted into such a graph representation using axiom patterns [25]; for example, the edge between *MET* and *MAPK3* could arise from an axiom $MET \sqsubseteq \exists \mbox{ activates}.MAPK3$ and the edge between *FOXP2* and *GO:0071625* from the axiom $FOXP2 \sqsubseteq \exists \mbox{ hasFunction }.\mbox{GO:0071625}$. The dashed edge between *FOXP2* and GO:0044708 is an edge that would be generated through entailment based on these axioms.

## Semantic similarity

In many biomedical and computer science applications it is useful to determine how similar two concepts are. Measures that compute similarity between concepts are semantic similarity measures, and semantic similarity measures have received renewed interest recently with the development of novel methods based on representation learning [31, 32]. Semantic similarity measures are used to compare words and terms in natural language texts [32], entities represented in graphs and knowledge graphs [33–36] and ontology classes based on the knowledge within the ontologies [37].

Semantic similarity measures can be used as unsupervised methods for association prediction, as features in supervised learning models or in clustering algorithms. Ontology-based similarity measures have been applied to a variety of tasks such as predicting protein–protein interactions [38–41], gene–disease associations [42–44], diagnosing patients [45–47], determining sequence similarity [48] or evaluating computational methods which predict ontology class annotations [49].

In ontologies, we can compute semantic similarity between classes, individuals and annotated entities. A function $sim: D \times D$ is a similarity function on a domain $D$ if it is non-negative ($sim(x, y) \leq 0$), symmetric ($sim(x, y) = sim(y, x)$) and if self-similarity yields the highest similarity values within the domain ($sim(x, x) = \max _{D}$), or—as a weaker version—if self-similarity is higher than similarity to any other domain entity ($sim(x, x)> sim(x, y)$) [50].

A simple similarity measure, $sim_{Rada}$, can be based on the shortest path between two nodes in the graph [51]. It can be defined as

$$\begin{align*} & sim_{Rada}(x, y) = \frac{1}{dist_{SP}(x, y) + 1}. \end{align*}$$

**Figure 2:**
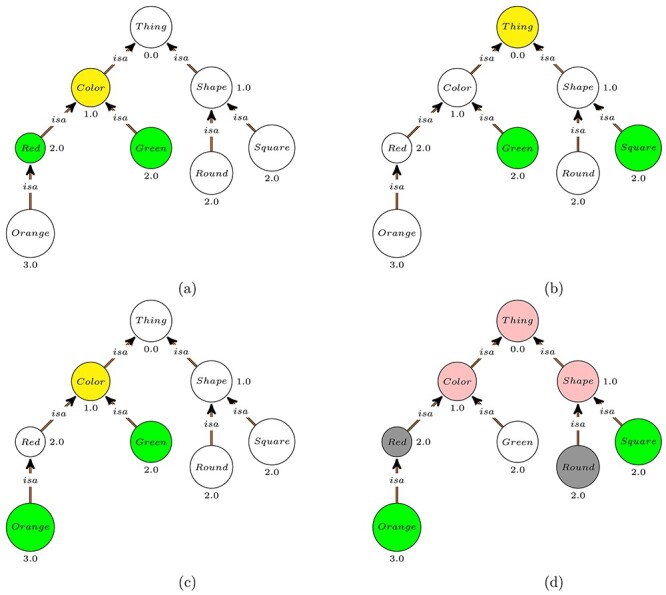
A fragment of the PATO ontology focusing on colors and shapes. Numbers near classes indicate the specificity of the classes.

This similarity measure is useful when edges in a graph correspond mostly uniformly to some kind of semantic distance. However, when comparing ontology classes, edges represent axioms involving two classes which may not correspond to this assumption. For example, is-a edges order classes from general to more specific, such as in the ontology in Figure 2a. In this figure, $sim_{Rada}(Color, Shape)$ will have the same value as $sim_{Rada}(Red, Green)$ since these two classes have the same distance in the graph. However, in many applications $Red$ and $Green$ should be more similar than $Color$ and $Shape$ because they are both colors. In this case, distance-based similarities might not be very intuitive and a measure of class *specificity* needs to be considered.

There are many ways to compute class specificity. For instance, we can compute specificity as a function of the depth, number of children or the information content of a class. Formally, class specificity is a function $\sigma : C \mapsto \mathbb{R}$ which meets the condition that for all $x, y \in C$, if $x \sqsubseteq y$ then $\sigma (x) \geq \sigma (y)$ [52]. The specificity measure can be defined using only the classes within an ontology (such as measures that consider the number of super-classes a class has, or the distance of a class to the root), or using information such as the number of instances of a class, or the number of annotations of a class within a database.

One widely used methods to determine class specificity is the Resnik measure [53], which defines the specificity of a class as its information content:

$$\begin{align*} & IC_{Resnik}(x) = -\log p(x), \end{align*}$$

where

$$\begin{align*} & p(x) = \frac{|I(x)|}{|I(\top)|} \end{align*}$$

and $I(x)$ is the set of instances of $x$ (or the set of annotations of a class within a database).

Overall, a large number of semantic similarity measures have been developed [52]. Pairwise similarity measures compute the similarity value between two classes. Examples of pairwise similarities used in the biomedical field include Resnik’s [53], Lin’s [50], Jiang & Conrath’s [54] and Schlicker’s [42] similarity measures. Many of these measures are variations of the Resnik measure which defines the similarity between classes $x$ and $y$ as the information content of their *most informative common ancestor (MICA)*:

$$\begin{align*} & Sim_{Resnik}(x, y) = IC(MICA(x, y)). \end{align*}$$

In the example in Figure 2a, $Sim_{Resnik}(Red, Green)$ is equal to $1.0$ and $Sim_{Resnik}(Color, Shape)$ is equal to $0.0$ although they have the same distance. The downside of this similarity measure is that it does not take into account the specificity of the compared classes and all classes under the same MICA will have the same similarity value. For instance, in Figure 2b $Sim_{Resnik}(Green, Square)$ is equal to $0.0$ which is the same as $Sim_{Resnik}(Color, Shape)$ and in Figure 2c $Sim_{Resnik}(Red, Green)$ and $Sim_{Resnik}(Orange, Green)$ are both equal to $1.0$. To solve this issue, Lin’s measure [50] also considers information content of the compared classes:

$$\begin{align*} & Sim_{Lin}(x,y) = \frac{2\cdot IC(MICA(x,y))}{IC(x) + IC(y)}. \end{align*}$$

With this measure, $Sim_{Lin}(Red, Green)$ is equal to $0.5$ whereas $Sim_{Lin}(Orange, Green)$ is equal to $0.4$ which is more intuitive.

When comparing two instances of ontology classes, or two entities annotated with classes in an ontology, we usually need to compare sets of classes. For example, we would have to compute the similarity of the set of all Gene Ontology annotations of one protein with the set of all Gene Ontology annotations of a second protein. There are two ways of determining the similarity between two sets of classes $A$ and $B$. First, we can compute the pairwise similarities between all pairs of classes $(a,b)$ such that $a \in A$ and $b \in B$, and then combine similarity values according to some combination strategy (such as computing the average). Second, we can directly define a similarity measure between the two sets $A$ and $B$ using a set similarity measure. For instance, we can use the Jaccard index between the two sets:

$$\begin{align*} & Sim_{Jaccard}(X, Y) = \frac{|X \cap Y|}{|X \cup Y|}. \end{align*}$$

To make this a semantic similarity, we would at least close each of the sets $X$ and $Y$ with respect to superclass axioms, i.e. if $C \sqsubseteq D$ and $C \in X$ then $D \in X$. Figure 2d depicts the propagation of ontology classes for computing the similarity between a square-and-orange thing and a round-and-red thing. Set similarity can also incorporate class specificity, such as the weighted Jaccard index in the SimGIC [55] measure.

Semantic similarity measures have a variety of applications and a large number of software packages have been developed to ease their use [56]. One prominent example is the Semantic Measures Library [57] which is a comprehensive Java library that allows to compute hundreds of different semantic similarity measures.

A common problem of semantic similarity measures is that it is difficult to choose the right measure for a particular application. Similarity measures behave differently depending on their applications. For example, using different similarity measures to predict PPIs will result in different performance [37, 55] depending on the organisms. Similarity measure are also not immune to biases in data and different similarities may react to the biases differently [44, 58]. Furthermore, they are hand-crafted measures that are not able to adapt automatically to the underlying data or application.

## Embedding ontologies

Another option to define similarity measures on ontologies is through the use of embeddings. An embedding is a structure-preserving map from one mathematical structure to another. The idea behind using embeddings is that the second structure may enable different or additional operations which are not possible in the first structure. For example, if we take ontologies or graphs that are discrete entities and map them into a continuous space (or real-valued vector space), we can apply machine learning or continuous optimization algorithms which operate on continuous data; there are also natural similarity measures between real-valued vectors such as the cosine similarity or other distance measures and metrics.

While there are many structures in which ontologies may be embedded (such as embedding axioms within the natural numbers so that proofs by diagonalization become possible [59, 60]), we are mainly interested in embedding ontologies within real-valued vector spaces so that we can apply modern optimization and machine learning algorithms. The key question when embedding ontologies is which structure (of the ontology) to preserve within ${\mathbb{R}}^n$ and under which operations in ${\mathbb{R}}^n$ this structure is preserved. We classify approaches of embedding ontologies in three main types, based on what aspects of the ontologies are preserved in ${\mathbb{R}}^n$. First, there are graph-based approaches which treat ontologies as graphs similar to how ontologies are treated by many semantic similarity measures, and the embeddings preserve this graph structure within ${\mathbb{R}}^n$. Second, *syntactic* approaches treat axioms similar to sentences and preserve syntactic regularities (such as frequencies of co-occurrences) in ${\mathbb{R}}^n$. Third, we consider *model-theoretic* approaches which preserve model-theoretic properties within ${\mathbb{R}}^n$ as a part of the embedding.

### Graph-based ontology embeddings

Graph-based embedding methods preserve a graph structure within ${\mathbb{R}}^n$. One form of graph embeddings is based on random walks. In these methods, graphs are generated from ontologies using the methods we described in Section 2 (Fundamentals: axioms, graphs and knowledge graphs), then random walks are used to explore the neighborhood of each node in the graph, and finally the set of walks is used as the basis of the embeddings.

One of the first methods for learning graph embeddings through random walks was DeepWalk [61] which generates a corpus of sentences (i.e. sequences of nodes in the graph) through random walks starting from each node in the graph, and then applies Word2Vec [32] on the resulting corpus to obtain embedding vectors; the embeddings generated by Word2Vec preserve co-occurrence relations within a context window. DeepWalk can also be extended to include labeled edges and be applied to knowledge graphs [62]; walk-based methods have been used to embed graphs generated from ontologies [26, 63] or combinations of knowledge graphs and ontology-generated graphs [64].

For example, for the graph in Figure 1, the random walks can generate sentences such as

- *FOXP2 cooex ST7 hasFunction GO:0044708...*
- *FOXP2 hasFunction GO:0071625 is-a GO:0044708...*

and Word2Vec will then embed each node and edge label while preserving co-occurrence relations within this corpus [65]. Node2Vec [66] is a modified model that does explore the original graph through biased random walks and therefore can force walks to remain within a certain distance of the origin node, or explore further away, depending on the choice of a parameter. OWL2Vec uses the biased random walks from Node2Vec to embed graphs generated from axioms [26].

Random walks have long been used as a model that simulates diffusion of information within a network [67–69] and can be used to identify and score node importance. In graph embeddings, these walks explore node neighborhood and generate a ‘linear’ representation (i.e. sequences of symbols); the walks account for graph structure such that nodes that are reached more often by a random walk also occur more often in the resulting corpus (and co-occur more often with the original node). Word2Vec, as a model that embeds sequences of symbols while maintaining this co-occurrence [65], generates embeddings that maintain this syntactic structure within the walks, and therefore aspects of the graph structure as well. Furthermore, some of the semantics of the axioms in the ontology can be encoded as constraints on the random walks or encoded in the graph; for example, symmetry can be modeled as a bi-directional edge, disjointness as a ‘barrier’ preventing a walk’s transition, etc. It is obvious that the graph that is generated from the ontology axioms, and the information it captures, is crucial for generating useful embeddings [26].

Translational embeddings methods are a family of representation learning methods on knowledge graphs which model relations in the knowledge graph as translation operations between graph node embeddings. The methods have been successfully applied for several tasks such as link prediction, knowledge-graph completion and others. The methods represent knowledge graphs as a set of edges $(s, p, o)$ (triples) and define a translation operation which translates $f_\eta (s)$ to $f_\eta (o)$ depending on the relation $p$. Here, $f_\eta $ is a graph embedding.

TransE [70] was an early translational embedding method. It uses a vector representation for relations that have the same dimensions as vectors representing nodes, and defines the translation operation as the addition of the relation vector to the node vector:

$$\begin{align*} & f_\eta(s) + f_\eta(p) \approx f_\eta(o) \end{align*}$$

and further defines a scoring function for an edge based on the translation operation:

$$\begin{align*} & f_{sc}(s, p, o) = \left\lVert f_\eta(s) + f_\eta(p) - f_\eta(o)\right\rVert. \end{align*}$$

Then, it minimizes the following loss function to learn $f_\eta $:

$$\begin{align*} & \sum_{(s, p, o) \in KG} \sum_{(s^{\prime}, p, o^{\prime}) \in KG^{\prime}} \big[\gamma + f_{sc}(s, p, o) - f_{sc}(s^{\prime}, p, o^{\prime})\big]_+, \end{align*}$$

where $KG^{\prime}$ is a set of negative or corrupted triples that are not in the graph, $\big [x\big ]_{+}$ indicates the positive part of $x$ and $\gamma $ is a hyperparameter. This model can only accurately represent one-to-one relations and it is not suitable for one-to-many and many-to-many relations while in graphs generated from ontologies, even when focusing only on the subclass hierarchy, there are many such relations. Furthermore, TransE does not support transitive, symmetric or reflexive relations which are all important for faithfully embedding ontologies.

Many TransE successors have been developed to overcome the original model’s limitations. For example, TransH [71] extended TransE by moving the translation operation to a relation-specific hyperplane. TransH represents each relation by two embedding vectors, the norm vector of the hyperplane (denoted as a function $w_\eta $) and a translation vector (denoted as a function $d_\eta $). The scoring function is then defined as follows:

$$\begin{align*} & \begin{split} f_{sc}(s, p, o) = & \lVert(f_\eta(s) - w_\eta^{\top}(p)f_\eta(s)w_\eta(p)) + d_\eta(p) - \\ & (f_\eta(o) - w_\eta^{\top}(p) f_\eta(o) w_\eta(p))\rVert. \end{split} \end{align*}$$

With an additional vector, TransH performs translation operation on an augmented hyperplane and can therefore model one-to-many and many-to-many relations better than TransE. There are also many other models with various advantages and disadvantages [33, 34].

Some translational models are specifically designed to capture some properties of ontologies such as hierarchical relation. On2Vec [72] embeds taxonomic relations by adding an additional scaling parameter to the loss function so that embeddings of more specific classes are in closer proximity than embeddings of more general classes, i.e. the class embeddings converge along the subclass hierarchy. JOIE [73] embeds instances and ontology classes using an ontology hierarchy loss function in which hierarchical relations between classes are embedded based on a non-linear transformation of the subclass into the superclass. TransC [74] and TransFG [75] embed classes as regions instead of points within ${\mathbb{R}}^n$ and define hierarchical relations between classes as relations between the regions in which they are embedded; for example, if $C$ is a subclass of $D$, the manifold which is the embedding of $C$ should lie within the manifold corresponding to $D$.

Translational embeddings are able to explicitly capture the graph structure and preserve some interpretability through the use of vector operations; however, they cannot always capture axioms such as transitivity, symmetry or reflexivity of relations. Furthermore, any graph-based method will focus on a set of axioms that are encoded by graph patterns and lose some information that is not captured by these patterns; many ontological axioms such as disjointness and axioms involving combinations of different logical operators often cannot be fully converted to a graph.

### Syntactic approaches

Ontologies in OWL format provide a structured representation of biological knowledge in the form of logical axioms, and not all the axioms in an ontology can be represented naturally in a graph; this limits the ability of these methods to utilize all information encoded in the ontology. Syntactic embeddings embed ontologies in ${\mathbb{R}}^n$ considering only the set of axioms without creating an intermediate graph-based representation.

Onto2Vec [76] is a method that generates embeddings for ontology classes and instances taking into account the logical axioms that define the semantics of ontology classes. Onto2Vec takes an ontology $O$ as input, uses an automated reasoner to entail additional logical axioms, mainly subclass axioms between named classes; it then treats each asserted or inferred axiom as a sentence and embeds the set of axioms using the Word2Vec language model. This allows Onto2Vec to embed ontologies directly based on their axioms while considering all axiom types, no matter how complex they are.

OPA2Vec [77] extends Onto2Vec to not only include logical axioms but also OWL annotation properties as well. OWL annotation properties in ontologies relate classes and relations to their labels, synonyms, definitions, and other types of information. OPA2Vec combines the corpus generated from the asserted and entailed logical axioms in Onto2Vec with a corpus generated from selected annotation properties (or all annotation properties). For example, from the annotation assertion that an OWL class $C$ has a label $L$ (using the rdfs:label annotation property in the OWL annotation axiom), OPA2Vec generates the statement C rdfs:label L, using the complete identifier for $C$ and rdfs:label, and expressing $L$ as a string literal; for instance, the annotation assertion of the class *Nuclear periphery* ( GO:0034399) and its label is expressed as the sentence “<http://purl.obolibrary.org/obo/GO_0034399> <http://www.w3.org/2000/01/rdf-schema#label> nuclear periphery”. The identifier for the class $C$ occurs within the ontology axioms and obtains parts of its meaning through the axioms; to ensure that the natural language terms used in the annotation properties have their ‘natural’ meaning as used in biomedical texts, OPA2Vec uses transfer learning and pre-trains a Word2Vec language model on biomedical literature texts, and then updates the model to generate the embeddings from the axioms plus annotation property assertions.

### Model-theoretic or semantic

None of the embedding methods discussed so far are semantic in the sense that they use the semantics of the underlying logic (as discussed in Section 2). Instead, the embedding methods are based on syntactic co-occurrences or preserving certain graph properties. However, the main advantage of using languages with an explicit semantics is that they provide constrains on how symbols should be interpreted.

EL Embeddings [78] aim to embed ontologies by mapping the symbols in the ontology into one specific interpretation, i.e. the embedding is identical to, or approximates, the interpretation function $\mathcal{I}$ discussed in Section 2. Given an ontology $O$, let $\Sigma (O)$ be the class, relation, and instance symbols in $O$. EL Embeddings find an embedding that maps $\Sigma (O)$ into ${\mathbb{R}}^n$, $f_e: \Sigma (O) \mapsto{\mathbb{R}}^n$ such that $f_e(\Sigma (O))$ is an interpretation of the axioms in $O$ (i.e. all axioms in $O$ are true in $f_e(\Sigma (O))$, $f_e(\Sigma (O)) \models O$). Such an embedding yields a faithful representation of logical operators and quantifiers.

Formally, EL Embeddings embed classes as $n$-balls in $n$-dimensional space and relations as $n$-dimensional vectors. The correspondence with the semantics of the axioms in the ontology is established by setting the domain of discourse to ${\mathbb{R}}^n$ and the following condition: for all classes $C \in \Sigma (T)$ and relations $r \in \Sigma (T)$ it defines $f_e(C) = C^{\mathcal{I}}:$

$$\begin{align*} & C^{\mathcal{I}} = \{ x \in \mathbb{R}^n | \left\lVert f_e(C) - x \right\rVert < r_e(C) \}, \end{align*}$$

where $r_e(C)$ is the radius of the $n$-ball that corresponds to $C$, and $f_e(r) = r^{\mathcal{I}}:$

$$\begin{align*} & r^{\mathcal{I}} = \{ (x,y) | x + f_e(r) = y \} \end{align*}$$

The latter condition is similar to the TransE translation operation applied to instances.

The embeddings are generated through optimization using a set of loss functions that correspond to different normal forms of the axioms in ontologies; such normal forms can be generated for ontologies formalized in the Description Logic EL [79], but may not exist for other, more expressive logics. Using these embeddings it is possible to approximate the intended semantics of the language within the embedding space. In particular, it can be shown that if the loss can be reduced to zero, the resulting embedding corresponds to a model of the ontology [78]. Similar approaches to EL Embeddings are also investigated for querying knowledge graphs using logic formulas [80].

### Using embeddings as semantic similarity measures and in machine learning methods

Embeddings can generate (distributed) representations of the symbols in ontologies while preserving syntactic or semantic properties. These representations—vectors in ${\mathbb{R}}^n$—can be visualized using dimensionality reduction techniques such as principal component analysis or tSNE [81]. They can also be used to compute similarity using any kind of similarity or distance measure applicable to real-valued vectors, in particular the cosine similarity or the Euclidean distance. For example, ontology embeddings generated for proteins based on their associations with classes in the Gene Ontology can be used to determine whether two proteins are (functionally) similar; this functional similar can then be used to predict interactions between proteins based on the hypothesis that similar proteins are more likely to interact [37, 82].

Another useful applications of ontology embeddings is as a part of machine learning models in which either a single embedding is used as input or multiple embeddings are used as input. Single embeddings can be used in classification and related tasks. Given an ontology $O$ with individuals $I$, classes $C$ and relations $R$, a (binary) classification function is a function $c: I \cup C \cup R \mapsto \{0,1\}$, and the task of a machine learning model is to approximate $c$. Since $I$, $C$ and $R$ consist of symbols, machine learning algorithms that approximate $c$ need to approximate functions from symbols into $\{0,1\}$. Alternatively, using vector space embeddings $f_e: I \cup C \cup R \mapsto{\mathbb{R}}^n$, the classification function $c$ approximated by a machine learning model will be a different function: $c: {\mathbb{R}}^n \mapsto \{0,1\}$; overall, the objective to optimize will be based on the combined function $c \circ f_e$. The introduction of $f_e$ as part of such problems has several advantages. First, while the vocabulary of $O$ may be large and consist of thousands of class, relation and individual symbols, $f_e$ usually embeds these entities in a space of relatively small size (depending on the chosen parameter $n$); the embedding preserves certain structural characteristics of the ontology $O$ similar to a ‘module’ [83] in the ontology, thereby making this local information available to an optimization algorithm that finds $c$; and embeddings in ${\mathbb{R}}^n$ allow gradient descent methods to be applied directly which are used in many modern machine learning methods.

Machine learning can also be used to approximate functions that take more than one embeddings as input, and these functions can then be used to predict relations between the entities that were embedded [34], or to learn similarity measures between ontology entities. For example, if the similarity between two protein embeddings is supposed to be a measure of whether or not they interact, using a set of proteins that interact can be used to learn a function that predicts, given two embeddings as an input, whether the proteins they represent should interact. Many neural network architectures and other machine learning models can be used for this task; architectures that are used for similarity learning, such as Siamese neural networks, seem to perform well in practice [63].

Machine learning with embeddings generated from ontologies has been used successfully in several biological applications, including classifying genes and genetic variants into cancer driver and non-driver genes/variants [84], detecting (causative) relations between genes and diseases based on comparing phenotypes (and other ontology-based features) [63, 77], predicting PPIs, as well as identifying drug–target interactions [85].

## Ontologies as constraints

Ontologies embeddings are a useful technique to make information in ontologies available as background knowledge to define similarity measures or to learn features for machine learning models. In these cases, ontologies are used as the *input* of a similarity function or a machine learning model. However, ontologies can also be used as an *output* of a machine learning model and the axioms in the ontology used to constrain the output of a function, such as in the case when determining if the predictions of a machine learning model are consistent with the axioms in the ontology, or the aim of the model is to predict associations of an entity with ontology classes.

Ontologies are used as structured output in many domains in which the primary task is to predict whether some entity has a relation with one or more ontology classes, such as predicting genotype–phenotype relations (using phenotype ontologies as output), predicting gene–disease or drug–disease associations (using disease ontologies as output), or predicting protein functions (using the Gene Ontology as output). At the very least, these tasks need to satisfy the hierarchical constraints imposed by the ontologies in the output space: if an entity $e$ is predicted to be associated with a class $C$, and that class $C$ is a subclass of $D$, then $e$ must also be associated with $D$. Similar constraints arise from other axioms in the ontology [15].

In general, there are at least five different approaches to using hierarchical relationships as constraints in classification models: flat, local per node, local per parent, local per level, and global hierarchical classification [86]. Flat classification is when the hierarchical constraints are not used during the prediction or training and the classification is done only using individual classes, and the consistency with the hierarchical constraints is enforced by propagating scores along the hierarchy only after predictions are made. This approach employs the constraints imposed by the ontology independently from the training or prediction process. In a local per node setting, a binary classifier is built for every class and predictions are made starting from the most general classes first and then moving to more specific ones, and stopping the prediction process once classes are predicted as negative. In a local per parent and local per level setting, multi-class classifiers are used for children classes of a parent or classes at the same level, respectively. Similarly to local per node classifiers, the prediction is performed in a step-wise manner from the most general class to more specific ones, and terminated once predictions are negative. The main drawback of local classifiers is that all classification models are trained independently from each other, and during the prediction process errors will propagate from general classes to more specific ones. Global hierarchical classifiers include the hierarchical constraints during training of a machine learning model, either as soft constraints or hard constraints, and also during prediction so that the output labels are forced to be consistent with the ontology axioms. The advantages of these models are that they take the semantics into account during training and therefore potentially reduce the search space; and that they can exploit dependencies between classes during training and prediction; the disadvantage is often the increased complexity of these classifiers [86].

While hierarchical machine learning models are used across many different application domains, life science ontologies are standing out with their large size and complex set of axioms; it is no surprise that constrained optimization methods applicable to large ontologies have emerged from research in bioinformatics. In particular prediction of functions and phenotypes benefits from machine learning models that are constrained by ontologies, as the goal of these methods is to predict the associations of a gene or gene product with a set of classes in an ontology.

The long-running Computational Assessment of Function Prediction (CAFA) challenge [49] evaluates the state of the art of computational function and phenotype prediction methods in regular intervals, and has also established evaluation measures for measuring performance of predictive models which consider some of the axioms in an ontology [87]. CAFA is also one of the drivers in developing novel ontology-based prediction methods. The key challenge of function prediction related to ontologies and constraints is the large number of classes; with over 40,000 classes in the Gene Ontology, and a protein potentially having any combination of these classes as functions, there is a lower bound of over $9.8 \times 10^{4059}$ possible combinations of functions a protein may have while remaining consistent with the Gene Ontology [88]; it is clear that novel methods must be investigated to reduce the size of this problem space.

Some computational function prediction methods do not use information about the ontology during training or prediction but combine and propagate information afterwards [89–91]; others use hierarchical top-down prediction methods in which the subclass hierarchy of the ontology is exploited [92]. There are, however, dedicated machine learning methods that rely on the ontology structure during training and testing of the models. A structured Support Vector Machine (SVM) [93] generalizes an SVM by allowing different structures (sets, trees, graphs, sequences, etc.) as output. Structured SVMs utilize similarity measures between the structured objects (such as tree similarity or graph similarity) in loss functions, and use a cutting plane method [94] to significantly reduce the number of constraints that need to be examined. Structured SVMs have been applied both to the prediction of functions based on the Gene Ontology [95] and phenotypes based on the Human Phenotype Ontology [96].

More recently, the focus in function prediction has been on using different neural network architectures that use ontologies as part of their structure or optimization objective. In the context of function prediction using neural networks, research is primarily done on feature learning (or deep learning) with neural networks so that it becomes possible to recognize patterns in protein sequences that may be indicative of functions [16, 17]. However, several methods have also been developed that specifically incorporate the ontology structure as part of neural networks. One of the first such models was DeepGO [97] which used a convolutional neural network for feature learning and a constrained classifier based on the Gene Ontology for its predictions. Using sigmoid functions as classifiers, the DeepGO neural network classifier enforced that the output of the sigmoid classifier for a class $C$ would be less than the sigmoid output for any of its superclasses: if $C \sqsubseteq D$ then $\sigma (C) \leq \sigma (D)$. In DeepGO, this constraint is used both during training and prediction, thereby ensuring that classifications with DeepGO are consistent (with respect to the subclass axioms in the Gene Ontology), and also reducing the search space for an optimal solution. This hierarchical classifier significantly improved prediction performance when compared to “flat” classifiers that do not consider ontology structure. Further research added several improvements to the DeepGO classifier, both with respect to computational complexity [98] and by reformulating the ‘hard’ constraints implemented in DeepGO as “soft” constraints using Bayesian networks [99]. There are several further methods that incorporate hierarchical constraints in artificial neural networks [100–102], mostly using a variation of the methods employed by ontology-based predictors.

A related research direction uses ontologies to structure machine learning models themselves. A pioneering study used the Gene Ontology to form the structure of a neural network model which was then used to simulate cell growth based on genotype [103]. The resulting system, DCell [103], creates a small linear layer of neurons for each class in the Gene Ontology (or one of its subsets) and connects them according to the ontology’s subclass hierarchy. DCell not only makes predictions from genotype to growth phenotype; the direct correspondence that DCell establishes between an interpretable system like the Gene Ontology and the neural network architecture used for DCell’s predictions allows investigating which parts of the neural network were active in a prediction, and therefore generate hypotheses about the underlying biological processes and structures which are active on the pathways that lead from a genotype to phenotype. The DCell system shows how ontologies can be used to make the inner workings of neural networks “visible” and how ontologies can be used to turn blackbox prediction models into interpretable models. This correspondence can even be exploited in both directions; the mathematical relations between different parts of the DCell model have been used to find relations between biological systems that function like logic operators, i.e. parts of biological systems that behave in a Boolean manner [103], and predict epistatic interactions between complex genotypes involving three or more genes [104].

## Use case and application

Ontologies are used in almost every major biological database. There are more than $800$ ontologies in ontology repositories such as BioPortal [117] which are used to describe different biological and biomedical entities. Consequently, ontologies play a role in many different biomedical machine learning tasks such as genotype–phenotype association prediction [45, 46, 118], protein function prediction [49], drug–target prediction [119, 120], protein–protein interaction prediction [37, 48, 112], gene–disease association prediction [44, 121] and many others.

Here, we evaluate ontology embedding methods on the task of predicting interactions between proteins based on the hypothesis that functionally related proteins are more likely to interact. We demonstrate how different ontology embedding methods can be applied, and we provide Jupyter Notebooks for all our experiments at https://github.com/bio-ontology-research-group/machine-learning-with-ontologies. The software needed to reproduce all results as well as additional useful tools to develop predictive models with ontologies are summarized in Table 2.

**Table 2:** An overview of software tools and applications involved in computing semantic similarity and building machine learning methods with ontologies

Type	Method/tool	Description	URL
Processing and preprocessing ontologies	OWLAPI	Reference library to process OWL ontologies, supports most OWL reasoners [105]	https://github.com/owlcs/owlapi
	funowl	Python library to process OWL ontologies	https://github.com/hsolbrig/funowl
	owlready2	Python library to process OWL ontologies	https://pypi.org/project/Owlready2/
	Apache Jena	RDF library with OWL support	https://jena.apache.org/
	rdflib	Python RDF library with OWL support	https://github.com/RDFLib/rdflib
	Protégé	Ontology editor and knowledge engineering environment [106]	https://protege.stanford.edu/
Computing entailments, reasoning	ELK	Very fast reasoner for the OWL 2 EL profile with polynomial worst-case time complexity [107]	https://github.com/liveontologies/elk-reasoner
	HermiT	Automated reasoner supporting most of OWL axioms with exponential worst-case complexity [108]	http://www.hermit-reasoner.com/
	Pellet	OWL reasoner supporting most of the OWL constructs and supporting several additional features [109]	https://github.com/stardog-union/pellet
Generating graphs from ontologies	OBOGraphs	Syntactic conversion of ontologies to graphs, targeted at OBO ontologies	https://github.com/geneontology/obographs
	Onto2Graph	Semantic conversion of OWL ontologies to graphs, following the axiom patterns of the OBO Relation Ontology [110]	https://github.com/bio-ontology-research-group/Onto2Graph
Computing semantic similarity	Semantic Measures Library	Comprehensive Java library to compute semantic similarity measures over ontologies [57]	http://www.semantic-measures-library.org/sml/
	sematch	Python library to compute semantic similarity on knowledge graphs [111]	https://github.com/gsi-upm/sematch
	DiShIn	Python library for semantic similarity on ontologies [112]	https://github.com/lasigeBioTM/DiShIn
Embedding graphs	OWL2Vec	Method that combines generation of graphs from ontologies, random walks on the generated graphs and generation of embeddings using Word2Vec. Syntactically processes most OWL axioms [26]	https://github.com/oholter/matcher-with-word-embedings
	DL2Vec	Method that combines generation of graphs from ontologies, random walks on the generated graphs and generation of embeddings using Word2Vec. Syntactically processes most OWL axioms [63]	https://github.com/bio-ontology-research-group/DL2Vec
	Walking RDF&OWL	Method that combines generation of graphs from ontologies, random walks on the generated graphs and generation of embeddings using Word2Vec. Only considers the ontology taxonomy. [64]	https://github.com/bio-ontology-research-group/walking-rdf-and-owl
	RDF2Vec	Method to embed RDF graphs [62]	https://github.com/IBCNServices/pyRDF2Vec, https://github.com/dwslab/jRDF2Vec
	Node2Vec	Method to embed graphs using biased random walks [66]	http://snap.stanford.edu/node2vec/
	PyKEEN, BioKEEN	Toolkit for generating knowledge graph embeddings using several different approaches [113, 114]	https://github.com/SmartDataAnalytics/PyKEEN
	OpenKE	Library and toolkit for generating knowledge graph embeddings	https://github.com/thunlp/OpenKE
	PyTorch Geometric	Library for graph neural networks which can be used to generate graph embeddings [115]	https://github.com/rusty1s/pytorch_geometric
Embeddingaxioms	Onto2Vec	Embeddings based on treating logical axioms as a text corpus [76]	https://github.com/bio-ontology-research-group/onto2vec
	OPA2Vec	Embeddings that combine logical axioms with annotation properties and the literature [77]	https://github.com/bio-ontology-research-group/opa2vec
	EL Embeddings	Embeddings that approximate the interpretation function and preserve semantics for intersection, existential quantifiers and bottom [78]	https://github.com/bio-ontology-research-group/el-embeddings
Ontology-based constrained learning	DeepGO	Implements an ontology-based hierarchical classifier for function prediction. The hierarchical classification module is generic and can be used with other ontologies and applications [97]	https://github.com/bio-ontology-research-group/deepgo
	DEEPred	Automated Protein Function Prediction with Multi-task Feed-forward Deep Neural Networks [116]	https://github.com/cansyl/DEEPred
	DCell, Ontotype	Deep neural networks structured based on ontology axioms to enable interpretability and encode the biological structure of a cell within the neural network [103, 104]	http://d-cell.ucsd.edu/
	DeepMiR2GO	Inferring Functions of Human MicroRNAs Using a Deep Multi-Label Classification Model [102]	https://github.com/JChander/DeepMiR2GO

Proteins do not function in isolation, and many biological processes and functions are regulated by multiple proteins and their interactions. Databases such as String [122] collect information about PPIs from different sources with experimental evidence as well as PPIs that are computationally inferred and automatically assigned, and the functions of proteins are described using the Gene Ontology [15].

We created two PPI datasets, one for interactions occurring in humans and one for yeast, based on data from the String database [122]. We filtered out interactions with a confidence score less than $700$ to retain only high confidence interactions. Table 3 provides the total number of proteins and interactions in each dataset. We split the two datasets consisting of interaction pairs into train and test sets, with a ratio of 80% and 20%, respectively, and we used 20% of the training set as a validation set. We used these two datasets as benchmark sets for evaluating ontology embedding and semantic similarity methods, and we made the datasets with documentation publicly available for download and provided the links in our public repository so that anybody can use the same data to benchmark and compare ontology-based prediction methods; we intend to keep the benchmark updated when we become aware of new results. The training and validation sets should be used to train and tune model parameters and select the best models, while the evaluation results and comparisons should be reported using the test set.

**Table 3:** The total number of proteins and number of unique interaction pairs in training, testing, and validation datasets.

**Organism**	**Proteins**	**Total**	**Training**	**Validation**	**Testing**
Yeast	6,157	119,051	76,193	19,048	23,810
Human	17,185	420,534	269,143	67,285	84,106

We predicted PPIs based on the associations of proteins with their functions and cellular locations represented in the Gene Ontology [15, 123], known interactions between proteins and the axioms contained in the Gene Ontology. One key question is how to represent these three types of knowledge as axioms in an ontology or knowledge base. We adopted a representation scheme in which all entities (proteins, functions, cellular locations) are classes and the relations between the entities are expressed as axioms [30, 124]. Specifically, if there is an interaction between proteins $P_1$ and $P_2$, we asserted the axioms $P_1 \sqsubseteq \exists \mbox{interacts-with}.P_2$ and $P_2 \sqsubseteq \exists \mbox{interacts-with}.P_1$; if protein $P$ is associated with a Gene Ontology class $C$, we asserted the axiom $P \sqsubseteq \exists \mbox{has-function}.C$. We combined this set of axioms with the Gene Ontology (released on 22 February 2020) to form our knowledge base.

For graph-based embedding methods, we generated a graph by creating an edge for existential restrictions in subclass axioms: if $X \sqsubseteq \exists R.Y$ is an (asserted) axiom in the knowledge base (consisting of the Gene Ontology together with the axioms we added), we created nodes $X$ and $Y$, and an edge from $X$ to $Y$ labeled $R$. For the Onto2Vec and OPA2Vec embedding methods, we use the Gene Ontology together with the set of protein associations as input; Onto2Vec and OPA2Vec also compute the deductive closure of the resulting axioms (with respect to subclass axioms) and adds the entailed axioms to the knowledge base. The Jupyter Notebook data.ipynb in our repository provides source code to generate the datasets, the splits, and the input files for the different embedding methods.

We then generated ontology embeddings using EL Embeddings, Onto2Vec and OPA2Vec, and used the generated graph to produce embeddings through random walks, biased random walks (Node2Vec) and TransE. Only Onto2Vec and OPA2Vec use an automated reasoner to explicitly compute and add entailments while the other embedding methods either do not use any entailed axioms or generate and use them only implicitly. We then use these embeddings, as well as two semantic similarity measures (Resnik’s [53] and Lin’s [50]), to predict PPIs. For embeddings based on random walks, Onto2Vec and OPA2Vec, we use cosine similarity to compute the pairwise similarity of all pairs of proteins in our dataset, including the proteins in the training, test, and validation sets. TransE embeddings and EL Embeddings use a prediction function that not only depends on the embeddings of the entities but also the relation that should hold between them (see Section 4.1), and we use the prediction functions for interacts-with edges and compute a prediction score for all pairs of proteins in our dataset. We further use the semantic similarity measures to compute the similarity between all pairs of proteins. We use the similarity, or the values of the prediction functions for EL Embeddings and TransE, as predictions for an interactions between two proteins.

These predictions methods all rely exclusively on the embeddings generated and a similarity function between the embeddings (cosine similarity in most cases). However, the embeddings can also be used as part of a supervised machine learning model to predict the relations between protein; such an approach has the potential to improve the predictive performance results [76, 77], depending on the chosen machine learning model [63]. There are many possible machine learning methods and neural network architectures to use for these predictions and we cannot review all of them here. We only include a single example of using the ontology annotations in a Siamese neural network within the executable notebooks we provide. We train two models which use Gene Ontology classes as features represented as a binary vector and predict PPIs. The first model (SiameseNN in Table 4 and Table 5) uses classes that are specifically used in the annotations as input, and the second model (SiameseNN (Ont) in Table 4 and Table 5) adds all the superclasses and other related classes according to the true path rule in the Gene Ontology; both models use three dense layers of size 1024, 512 and 256 followed by the dot product and a sigmoid activation function to predict associations. Our results show that using the ontology structure improves performance of these predictions; furthermore, this model is the only one in which the prediction function itself is generated through machine learning while the other methods use a fixed similarity function; even without incorporating much of the ontology structure as features this approach performs well. Instead of binary vectors, a similar neural network architecture could be used with embedding vectors as inputs, and this approach can further improve the performance [77].

**Table 4:** Prediction performance for yeast PPIs. Best-performing results are highlighted in bold

Method	Raw Hits@10	Filtered Hits@10	Raw Hits@10	Filtered Hits@100	Raw mean rank	Filtered mean rank	Raw AUC	Filtered AUC
TransE	0.06	0.13	0.32	0.40	1125.4	1074.8	0.82	0.83
SimResnik	**0.09**	0.17	0.38	0.48	757.8	706.9	0.86	0.87
SimLin	0.08	0.15	0.33	0.41	875.4	824.5	0.84	0.85
SiameseNN	0.06	0.17	0.46	0.68	674.3	622.2	0.89	0.90
SiameseNN (Ont)	0.08	**0.19**	**0.50**	**0.72**	543.6	491.6	0.91	0.92
EL Embeddings	0.08	0.17	0.44	0.62	**451.3**	**394.0**	**0.92**	**0.93**
Onto2Vec	0.08	0.15	0.35	0.48	641.1	587.9	0.79	0.80
OPA2Vec	0.06	0.13	0.39	0.58	523.3	466.6	0.87	0.88
Random walk	0.06	0.13	0.31	0.40	612.6	587.4	0.87	0.88
Node2Vec	0.07	0.15	0.36	0.46	589.1	522.4	0.87	0.88

**Table 5:** Prediction performance for human PPIs. Best-performing results are highlighted in bold

Method	Raw Hits@10	Filtered Hits@10	Raw Hits@10	Filtered Hits@100	Raw mean rank	Filtered mean rank	Raw AUC	Filtered AUC
TransE	**0.05**	0.11	0.24	0.29	3960.4	3890.6	0.78	0.79
SimResnik	**0.05**	0.09	0.25	0.30	1933.6	1864.4	0.88	0.89
SimLin	0.04	0.08	0.20	0.23	2287.9	2218.7	0.86	0.87
SiameseNN	0.05	**0.15**	**0.41**	**0.64**	1881.10	1808.8	0.90	0.89
SiameseNN (Ont)	0.05	0.13	0.38	0.59	1838.31	1766.3	0.89	0.89
EL Embeddings	0.01	0.02	0.22	0.26	**1679.72**	**1637.7**	**0.90**	**0.90**
Onto2Vec	**0.05**	0.08	0.24	0.31	2434.6	2391.2	0.77	0.77
OPA2Vec	0.03	0.07	0.23	0.26	1809.7	1767.6	0.86	0.88
Random walk	0.04	0.10	0.28	0.34	1942.6	1958.6	0.85	0.86
Node2Vec	0.03	0.07	0.22	0.28	1860.5	1813.1	0.86	0.87

In the evaluation, for each protein $p$ we rank all other proteins $p_i$ based on their similarity (or the value of the prediction function) to $p$. We then consider positives as pairs $(p, p_k)$ which are PPIs included in our test set, and we report hits (recall) at ranks $10$ and $100$, mean rank at which the PPIs are found, and the ROCAUC (using micro-averages per protein). Results are separated in *Raw* and *Filtered*; *Raw* results evaluate all pairs of proteins while *Filtered* results evaluate all pairs of proteins except the pairs that are included in the training or validation sets. *Filtered* results are usually better since training pairs are not considered in the evaluation. We made Jupyter Notebooks available for all our experiments, and Table 4 summarizes the results for yeast and Table 5 for human; all results in these tables can be reproduced using the Jupyter Notebooks.

Overall, while our results are by no means a comprehensive evaluation and are limited to the task of predicting PPIs, and most of the methods we use rely on unsupervised methods, we can obtain some information from our experiments. Traditional semantic similarity measures, in particular Resnik’s measure [53], perform well across many evaluations, in particular in recall at the first ranks, and often has better performance than ontology embedding methods combined with cosine similarity; this property has also been observed in other applications where Resnik’s measure performs better than most other unsupervised methods [58, 76]. Moreover, exploiting more of the axioms generally yields better results as can be seen when comparing EL Embeddings with other embedding methods. Furthermore, exploiting longer, or more indirect, relations, either through random walks on graphs or through utilizing the semantics, usually improves results over methods that are based on local properties or simple adjacency.

While there is no ontology embedding method which is clearly superior to others, each of the methods has its own advantages and disadvantages. Representing ontologies as graphs leads to some loss of the information encoded in axioms which cannot be naturally represented in a graph. Syntactic embedding methods, on the other hand, have the advantage of being able to use all axioms in the ontology, including the ones which cannot be represented in a graph, and are not limited to particular axiom types or expressivity of the formal language. Semantic (model-based) embeddings such as EL Embeddings can use the model-theoretic semantics of formal languages but cannot easily be extended to new languages since they require specific loss functions to be designed which may prove challenging for some languages. An advantage of embeddings that rely on language models such as Word2Vec is that they can easily be combined with information in natural language, while different extensions for multi-modal embeddings exist for other methods [125] which usually require extending the model. Natural language texts can have information that is complementary to the structured information in ontologies, and combining structured information with text can often improve predictive model performance [126].

There are also different ways in which embeddings and ontologies can be used to predict associations, and they have different advantages and disadvantages as well. Semantic similarity measures determine similarity between entities in ontologies, are usually hand-crafted and interpretable. Ontology embeddings can be used with vector similarity measures such as cosine similarity, but interpretability is difficult to obtain as the embeddings are not generally invertible (i.e. given the image of an embedding, it is not possible to reconstruct its domain). Embeddings can also be used with additional machine learning algorithms to generate associations; these supervised methods usually perform better than using a fixed similarity measure as supervised learning can find functions that account for the specific features of the data and application of interest.

## Limitations and future work

Machine learning using ontologies involves a set of emerging techniques that have their roots in computer science and major applications in the life sciences where a large amount of ontologies have been developed and are applied to characterize data. Currently, several methods that allow background knowledge to be used by machine learning models are based on knowledge graphs and graph embeddings, and while these methods can be very successful, they lack the ability to utilize the model-theoretic semantics underlying ontologies. Ontologies, and representation artifacts based on similar formalisms, have the ability to represent more complex forms of knowledge, including using quantifiers, intersection, negation, and have the ability to represent inconsistent knowledge. Strong negation, for example, is crucial in constraining search and cannot be substituted with the limited form of negation that is sometimes applied in knowledge graphs (i.e. the closed world assumption in which facts that are not stated are considered false). However, while ontologies are able to express strong negation and other complex facts or rules, most ontology embedding methods are not yet able to adequately utilize them. Most syntactic and graph-based approaches do not interpret negation as constraints, or use any of the semantics associated with it, and can therefore not use negation to restrict search; and while model-based embeddings can utilize negation as part of the embedding they do not interact with the similarity measures or machine learning models that utilize the resulting embeddings.

Several approaches aim to systematically integrate symbolic representations and machine learning. Neuro-symbolic systems and neuro-symbolic integration [127, 128] provide a framework in which machine learning is integrated with symbolic representations; in the neuro-symbolic cycle, deductive inference is applied on the symbolic representations; embeddings project these representations into some space where they can be combined with data and where machine learning and optimization methods can be applied; and a knowledge extraction process maps the results back into the symbolic space. How to implement either of these projections is an active research area several of which we have reviewed here, and neuro-symbolic systems aims to put them together into a single framework. One crucial component in this cycle is the knowledge extraction which can be formulated as inverting an embedding (i.e. if $f_\eta $ is an embedding mapping the symbols $\Sigma (O)$ occurring in an ontology $O$ into ${\mathbb{R}}^n$, find $f_{\eta }^{-1}$ that maps from ${\mathbb{R}}^n$ into $\Sigma (O)$); while there are invertible linear embeddings into vector spaces [129, 130] they have not been explored in the context of symbolic representations such as ontologies. There is also recent interest in implementing the entire neuro-symbolic cycle, for example in vision [131]; however, with the rich set of formalized knowledge bases and the large amounts of data produced in the life sciences, we expect these systems to have major impact on how AI is applied in biology and biomedicine in the future.

Approaches to improve learning with ontologies while preserving and exploiting their semantics do not only include investigating embeddings into vector spaces (which, arguably, are mainly inspired by the needs of modern machine learning systems) but also approaches based on formal languages and logic, including Markov logic [132] and probabilistic inference [133]. Similarly, for extracting knowledge from data, new paradigms such as ‘reinforcement learning as inference’ [134] are increasingly being applied to generate explanations and representations that can be verified for consistency with background knowledge [135–137]. These methods significantly extend research that has been done in inductive logic programming and are another active area of research.

One main limitation of all the approaches we discussed here is their inability to consider quantitative information or data. In all cases, ontologies are used to model qualitative information and then possibly combined with other quantitative information after an embedding is generated; methods that can jointly learn on ontologies and quantitative information mapped to them include graph neural networks [138] which will likely see increasing adoption in the coming years.

Here, we reviewed methods that use ontologies as background knowledge to solve biomedical problems. The methods we reviewed range from semantic similarity over various forms of unsupervised feature learning (embedding) methods to constraining machine learning models using ontologies. These methods have in common that they use ontologies to solve biomedical problems that reside outside the domain of research on the ontologies themselves. However, there can be substantial methodological overlap with research on ontology engineering, ontology learning, quality control, querying, and reasoning with ontologies, as ontology embeddings can also be used for ontology alignment [139–141], as part of automated reasoning systems [142, 143] or to query knowledge bases [80, 144]. In the future, we expect to see even more integrated research on developing ontologies, ontology infrastructure and novel biomedical applications to which they can be applied.

**Key Points**

- Ontologies provide background knowledge that can be exploited in machine learning models.
- Ontology embeddings are structure-preserving maps from ontologies into vector spaces and provide an important method for utilizing ontologies in machine learning. Embeddings can preserve different structures in ontologies, including their graph structures, syntactic regularities or their model-theoretic semantics.
- Axioms in ontologies, in particular those involving negation, can be used as constraints in optimization and machine learning to reduce the search space.

## Data Availability

The data underlying this article are available in Github at https://github.com/bio-ontology-research-group/machine-learning-with-ontologies.

## Notes

**Maxat Kulmanov** is a postdoctoral researcher in computer science in the Bio-Ontology Research Group at King Abdullah University of Science and Technology. His research interests include knowledge discovery and data integration using artificial intelligence and Semantic Web technologies in biology and biomedicine.

**Fatima Zohra Smaili** is a doctoral student in computer science at King Abdullah University of Science and Technology. Her research focuses on ontology-based knowledge representation and machine learning.

**Xin Gao** is an associate professor in computer science, acting associate director of the Computational Bioscience Research Center and lead of the Structural and Functional Bioinformatics Group at King Abdullah University of Science and Technology. His research focuses on bioinformatics and machine learning.

**Robert Hoehndorf** is an associate professor in computer science and principal investigator of the Bio-Ontology Research Group at King Abdullah University of Science and Technology. His research focuses on combining knowledge representation and machine learning in biology.
